# To Hear or Not to Hear: Sound Availability Modulates Sensory-Motor Integration

**DOI:** 10.3389/fnins.2016.00022

**Published:** 2016-02-09

**Authors:** Ivan Camponogara, Luca Turchet, Marco Carner, Daniele Marchioni, Paola Cesari

**Affiliations:** ^1^Department of Neurological and Movement Science, University of VeronaVerona, Italy; ^2^Department of Architecture, Design and Media Technology, Aalborg University CopenhagenCopenhagen, Denmark; ^3^Department of Otolaryngology, University Hospital of VeronaVerona, Italy

**Keywords:** locomotion, auditory feedback, aftereffect, cochlear implant, footstep sounds

## Abstract

When we walk in place with our eyes closed after a few minutes of walking on a treadmill, we experience an unintentional forward body displacement (drift), called the sensory-motor aftereffect. Initially, this effect was thought to be due to the mismatch experienced during treadmill walking between the visual (absence of optic flow signaling body steadiness) and proprioceptive (muscle spindles firing signaling body displacement) information. Recently, the persistence of this effect has been shown even in the absence of vision, suggesting that other information, such as the sound of steps, could play a role. To test this hypothesis, six cochlear-implanted individuals were recruited and their forward drift was measured before (Control phase) and after (Post Exercise phase) walking on a treadmill while having their cochlear system turned on and turned off. The relevance in testing cochlear-implanted individuals was that when their system is turned off, they perceive total silence, even eliminating the sounds normally obtained from bone conduction. Results showed the absence of the aftereffect when the system was turned off, underlining the fundamental role played by sounds in the control of action and breaking new ground in the use of interactive sound feedback in motor learning and motor development.

## Introduction

During the past 20 years, the sensory-motor aftereffect after walking on a treadmill has been widely studied as a motor consequence resulting from a mismatch of information among the senses (Anstis, 1995; Durgin and Pelah, 1999; Durgin et al., 2005; Zanetti and Schieppati, 2007; Philbeck et al., 2008). Initially investigated by Anstis (1995), the sensory-motor aftereffect represents an unperceived forward displacement (usually called forward drift) which occurs while walking/running in place after having experienced several minutes of walking/running on a treadmill (Anstis, 1995; Durgin and Pelah, 1999; Durgin et al., 2005). Interestingly, the amount of forward displacement has been found to be proportional to the walking/running velocity on the treadmill and was assessed for different postures and tasks (Rieser et al., 1995; Durgin et al., 2005; Zanetti and Schieppati, 2007; Philbeck et al., 2008). The classical explanation of this effect is mainly related to a conflict between visual and proprioceptive information: during treadmill walking, while the proprioceptive receptors from the lower legs record movement, the visual system relays information that the body is still. Sensory information coming from the muscle spindles, in addition to tactile and vestibular receptors, combine to create a memory for these recent actions, which diverges from the information shared by the neural circuits responsible for the integration of afferent stimuli (Van Der Kooij et al., 1999). This mismatch of information leads to a perceptuo-motor re-calibration that can be seen as a forward displacement of the center of pressure while standing on a force plate (Zanetti and Schieppati, 2007), as a forward drift during walking in place (Anstis, 1995), or as an overshoot of a previously seen target (Rieser et al., 1995; Durgin et al., 2005). Although the information-conflict hypotheses between vision and proprioception are widely accepted, several studies demonstrated that the amount of forward drift was not reduced by a removal (Anstis, 1995; Durgin and Pelah, 1999; Durgin et al., 2005) or by a supplementation of optic flow (Durgin and Pelah, 1999). This suggests that the aftereffect might not be due to just proprioception and vision alone. Durgin and Pelah (1999) unexpectedly discovered that the forward displacement, measured in a running in place task after an adaptation period where participants were wearing earplugs, was double compared to the one measured when the earplugs were not employed (Durgin and Pelah, 1999). This led them to think that hearing vs. not hearing the steps while walking on the treadmill might change the perceptuo-motor calibration. In order to thoroughly test the influence of sound, Turchet and colleagues used a classic sensory-motor aftereffect paradigm which provided participants shoes that interactively delivered sounds simulating different ground materials, returning to the participant a vivid impression of walking in deep snow or on a floor made of concrete (Turchet et al., 2015). The authors showed that depending on the type of material experienced while walking on a treadmill, there was a different amount of unintentional forward drift while walking in place. A shorter forward drift was observed while “walking” in deep snow compared to “walking” on a solid surface; in fact, when walking in deep snow, participants experienced a clear sensation of sinking and higher effort (Turchet et al., 2010, 2013b, 2015; Turchet and Serafin, 2013). It is known that sounds influence movements particularly when there are no haptic or visual inputs (Dozza et al., 2007). Moreover, in a pathological situation, sounds can alleviate gait disturbances by regularizing the movement pattern (Rodger et al., 2014). In this regard, it has been shown in Parkinson patients that the injection of a sound of walking on gravel led to a low step length and coefficient of variation compared to when no sound was delivered (Rodger et al., 2014).

Since participants typically perform the sensory-motor aftereffect paradigm walking in place with their eyes closed, it might be that they relied principally on the sounds of their steps in order to perform the task successfully (Young et al., 2013; Cesari et al., 2014; Rodger et al., 2014) and regulated their movement pattern (Rodger et al., 2014). Hence, if sounds represent relevant feedback that humans rely on, it would be interesting to test the aftereffect in the total absence of sound. Up to now, no data is available having sounds “completely” removed. In normal hearing individuals, it is practically impossible to remove sounds completely due to the bones' conduction (Dauman, 2013). In order to test the sensory-motor aftereffect as “completely free from sounds,” we recruited hearing-impaired individuals with cochlear-implanted systems and tested their behavior with their system turned on and turned off. It is important to underline that by switching their system off, they were not able to receive any sound information, not even those transmitted by bone conduction, whereas their hearing was returned by simply switching their system on. In each hearing condition (having the system turned on or turned off), we asked participants to walk on a treadmill while they listened to their natural steps and while they listened to a simulation of them walking in deep snow (Turchet et al., 2010; Turchet, 2014). We quantified the amount of forward displacement in both hearing (system switched on and off) and both sound conditions (hearing the natural steps and hearing their steps as if performed on a surface covered by snow). In line with our previous findings, we expected a drift difference between the pre- and post-treadmill phases when the cochlear system was turned on, and a shorter forward drift when participants heard their steps as if walking in deep snow compared to hearing the actual sound of their natural footsteps. On the contrary, when the cochlear system was turned off, we expected no change in the amount of forward displacement between the pre- and post-treadmill phases.

## Materials and methods

### Participants

Six participants with a right mono-lateral cochlear implant (age range = 26.01 ± 9.79 years) were recruited for the experiment. Participants' data are represented in Table 1. None suffered from vestibular dysfunction. The experiment was approved by the ethical committee of the Department of Neurological, Biomedical and Movement Sciences at the University of Verona. All participants provided consent for their participation.

**Table 1:** **Participant data**.

**Participants**	**Gender**	**Years of implant**	**Etiology**	**Implantation site**
F.R.	M	9	Neurofibratosis type 2	Brainstem
D.M.	M	13	Trauma with bilateral cochlear fracture	Brainstem
M.A.	F	16	Congenital hearing loss	Cochlea
P.S.	M	15	Congenital hearing loss	Cochlea
M.G.	M	12	Congenital hearing loss	Cochlea
G.A.	F	16	Congenital hearing loss	Cochlea

### Apparatus

The apparatus consisted of a treadmill (HP/cosmos/Saturn 300/100r) and a laptop that delivered the footstep sound synthesis engine (Turchet, in press). This system was wirelessly connected to a pair of sandals augmented with pressure sensors (Turchet, 2014); a wired closed headphone set with a noise canceling system (Sennheiser PXC 450), and a motion capture system (Vicon MX) composed of eight infrared cameras set up to track an area of calibration made of 4 × 4 meters. Cameras were set to collect data at a sampling frequency of 100 Hz and the treadmill was positioned near the calibrated area. The total latency between the actual foot-to-floor contact and the synthesized sound heard was not noticeable since it amounted to about eight milliseconds (ms): three ms for the data acquisition and wireless transmission using the x-OSC wireless micro-controller board (Madgwick and Mitchell, 2013), 1 ms for the real-time data analysis, and 4 ms for the auditory feedback synthesis and delivery.

### Stimuli

Two sound stimuli were considered in this experiment: an artificial one, which consisted of interactively generated footstep sounds simulating an aggregate surface material as Snow-Sound (SS), and a natural one, where no additional auditory feedback was delivered and participants were hearing the actual sound of their footsteps as Natural Footstep Sound (NFS). The selection of the snow surface was inspired by our previous work showing that this simulated ground material was among those most easily recognized (Nordahl et al., 2010), and more importantly, because snow presents a high material compliance. The sound amplitude was set at 55.4 dB (A) measured with a phonometer CESVA SC-2c, whose microphone was positioned in the left and right speaker of the headphones. This value was selected according to the results of a previous experiment whose goal was to find the appropriate level of amplitude for synthesized sounds (Turchet and Serafin, 2013). This sound amplitude was effective in completely masking the actual footstep sounds produced by participants. The sonically simulated surface material was chosen to check the presence of expected pseudo-haptic illusions capable of altering the foot-haptic perception of hardness of both the treadmill platform and the carpeted laboratory floor (the two floors' hardness was similar). The experiment was conducted in a silent laboratory [background noise 46.7 dB (A)].

### Procedure

Participants were first asked to put on the sandals and wear the headphones. Subsequently, the experimenter placed six reflective markers on each foot for motion tracking. Specifically, these markers were placed bilaterally on specific anatomical landmark points (lateral malleolus, calcaneus, and 5th metatarsal head).

For each sound feedback (SS and NFS), participants underwent three experimental phases: control (C), exercise (E) and post-exercise (PE). This protocol was inspired by one previously reported (Zanetti and Schieppati, 2007; Turchet et al., 2015) and was a standard protocol for measuring the sensory-motor aftereffect (Anstis, 1995; Durgin and Pelah, 1999). In the control (C) phase, participants were asked to close their eyes and walk in place for 25 s. The exercise phase (E) consisted of walking for 3 min with their eyes open on the treadmill at a speed of 4.5 Km/h. Once the exercise phase ended, we asked participants to get off the treadmill and walk in place for 25 s. We named this phase Post Exercise phase (PE) (Anstis, 1995). Subsequently, they were asked to rest for at least 5 min before beginning the next trial (Zanetti and Schieppati, 2007; Turchet et al., 2015). The reason for asking participants to get off the treadmill after the exercise phase was due to the fact that the amount of drift could be ~1.5 m or more and it was objectively difficult to let participants perform the task while staying on the treadmill. Moreover, individuals performed the task with their eyes closed, and therefore safety was a consideration as well. During phases C, E and PE, the same sound stimulus was presented. For the C and PE phases, the experimenter touched the participant's shoulder at the beginning and at the end of the trial in order to inform him/her when to start and stop the task. During the E phase, the treadmill movement signaled the beginning and the end of the task. Individuals were free to select their own step frequency and step height. No indication was given about how to step in place.

Sound feedback was presented in a randomized order across participants, while the experimental phases were fixed. Each individual repeated the experimental phases twice: once with the Cochlear Implant on (CIon) and once with the Cochlear Implant off (CIoff). Before data collection, participants had the opportunity to become familiar with the shoes and the interactive sounds. They were not provided information about the type of material simulated by the synthesis model. Participants took, on average, about 1 h to complete the experiment. Fatigue was not an issue.

#### Questionnaire

At the end of the experimental data collection, subjects were asked to fill out a questionnaire and to answer by means of a Visual Analog Scale (VAS). The questionnaire was inspired by those previously utilized (Turchet et al., 2013a,b, 2015). For each sound condition, six questions were asked:

- [Effort] Evaluate the sense of effort you experienced while walking [0 = no effort, 10 = high effort]
- [Easiness] Evaluate the degree of easiness with which you walked while listening to the sounds [0 = very hard, 10 = very easy]
- [Sinking] Evaluate to what extent you had the impression that your feet were sinking into the ground [0 = not at all, 10 = very much]
- [Influence] Evaluate to what extent the sound influenced your way of walking [0 = not at all, 10 = very much]
- [Softness] Evaluate the impression of softness of the floor you walked upon. [0 = not soft at all, 10 = very soft]
- [Hardness] Evaluate the impression of hardness of the floor you walked on. [0 = not hard at all, 10 = very hard]

The order of presentation of the questions was randomized using a 6 × 6 Latin square. At the end of the questionnaire participants were asked to name the simulated surface material. The reported scores were then analyzed.

### Data handling

Motion capture signals were analyzed by means of Matlab R_2012a software. For each experimental condition, each experimental phase and type of auditory feedback, the participants' forward displacement (forward drift), step length and number of steps were considered for data analysis. The amount of forward drift was calculated by considering the displacement of the marker placed on the foot on the transversal plane. A moving average among points taken every 200 instants was considered (Turchet et al., 2015) and the total path length was calculated. For the step length, we first calculated the foot center by means of the Euclidean distance between the markers of the heel and the toe. We then calculated its first derivative and defined the start of the swing phase when the velocity of the foot's center point reached 5% of its maximum value. In this way, consecutive toe off were detected and the step length for each gait cycle was derived (the distance covered during each footstep in the transversal plane); the average of each step length in 25 s of walking was then computed. Numbers of steps were calculated by considering the number of toe off performed in 25 s.

### Statistics

In order to compare the effect of surface, we first entered all the calculated variables (step length, forward drift, number of steps) in a three-way repeated measure ANOVA, considering the Experimental Conditions (CIoff, CIon), Surface (SS, NFS) and Phase (C, PE) as within factors. Additional *t*-tests were performed for the questionnaire data, considering the two Surfaces (SS, NFS) in the CIon Experimental Condition for each of the six dependent variables (Effort, Easiness, Sinking, Influence, Softness, and Hardness). All *post-hoc* tests were performed using a Bonferroni correction (critical *p*-value = 0.05). Due to the small sample size, we checked for a normal distribution using the Shapiro-Wilk test and performed a parameter estimation. In the Shapiro-Wilk test, all the variables for each condition and phase showed a normal distribution (*p* > 0.05), while in the parameters estimation there was a significance level below 0.05.

## Results

### Kinematic results

The ANOVA for the variable Step Length showed no main effect or interactions. On the other hand, the Forward drift analysis showed a main effect for the Experimental Condition, [*F*_(1, 5)_ = 64.309, *p* < 0.001, η^2^ = 1], Surface [*F*_(1, 5)_ = 10.882, *p* = 0.022, η^2^ = 0.75], Phase [*F*_(1, 5)_ = 9.240, *p* = 0.029, η^2^ = 0.68], and an interaction between Experimental Condition X Phase [*F*_(1, 5)_ = 12.037, *p* = 0.018, η^2^ = 0.79].

**Figure 1:**
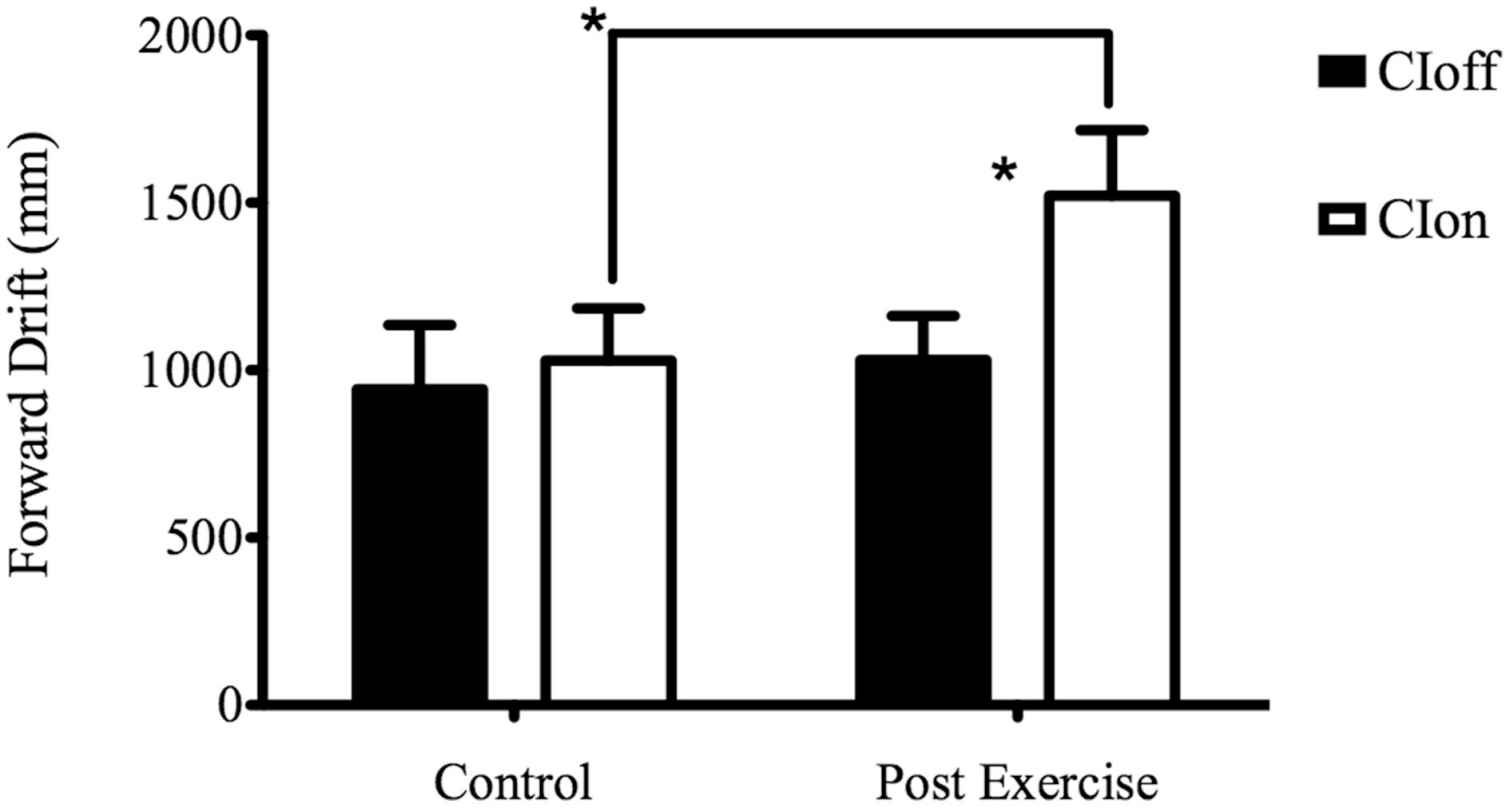
**Forward Drift for the interaction Experimental Condition X Phase**. ^*^Represents a level of significance of *p* < 0.05.

The *post-hoc* analysis for the Experimental Condition showed a higher forward drift for CIon compared to CIoff, demonstrating a reliance on sound feedback during the treadmill walking. The Surface factor unveiled a higher forward drift for the NFS compared to the SS; considering Phase, the ANOVA showed a higher value for the PE phase compared to the C phase. The interaction between Experimental Condition and Phase showed a higher value in PE compared to C only in the CIon condition, and a higher value for CIon compared to CIoff in the PE phase (see Figure 1). This corroborated the main effect found for the Experimental Condition in showing the absence of the aftereffect when no auditory information was available.

The ANOVA for the number of steps showed a main effect for Surface [*F*_(1, 5)_ = 62.500, *p* = 0.001, η^2^ = 1]; no other main effect or interactions were found. The *post-hoc* analysis showed a higher number of steps in the NFS condition compared to the SS condition.

We found a slightly significant interaction between Experimental Condition X Surface X Phase [F_(1, 5)_ = 5.96, *p* = 0.058, η^2^ = 0.50], which revealed a lower forward drift for SS compared to NFS in the C phase and only in the CIon condition. No other differences were found.

**Figure 2:**
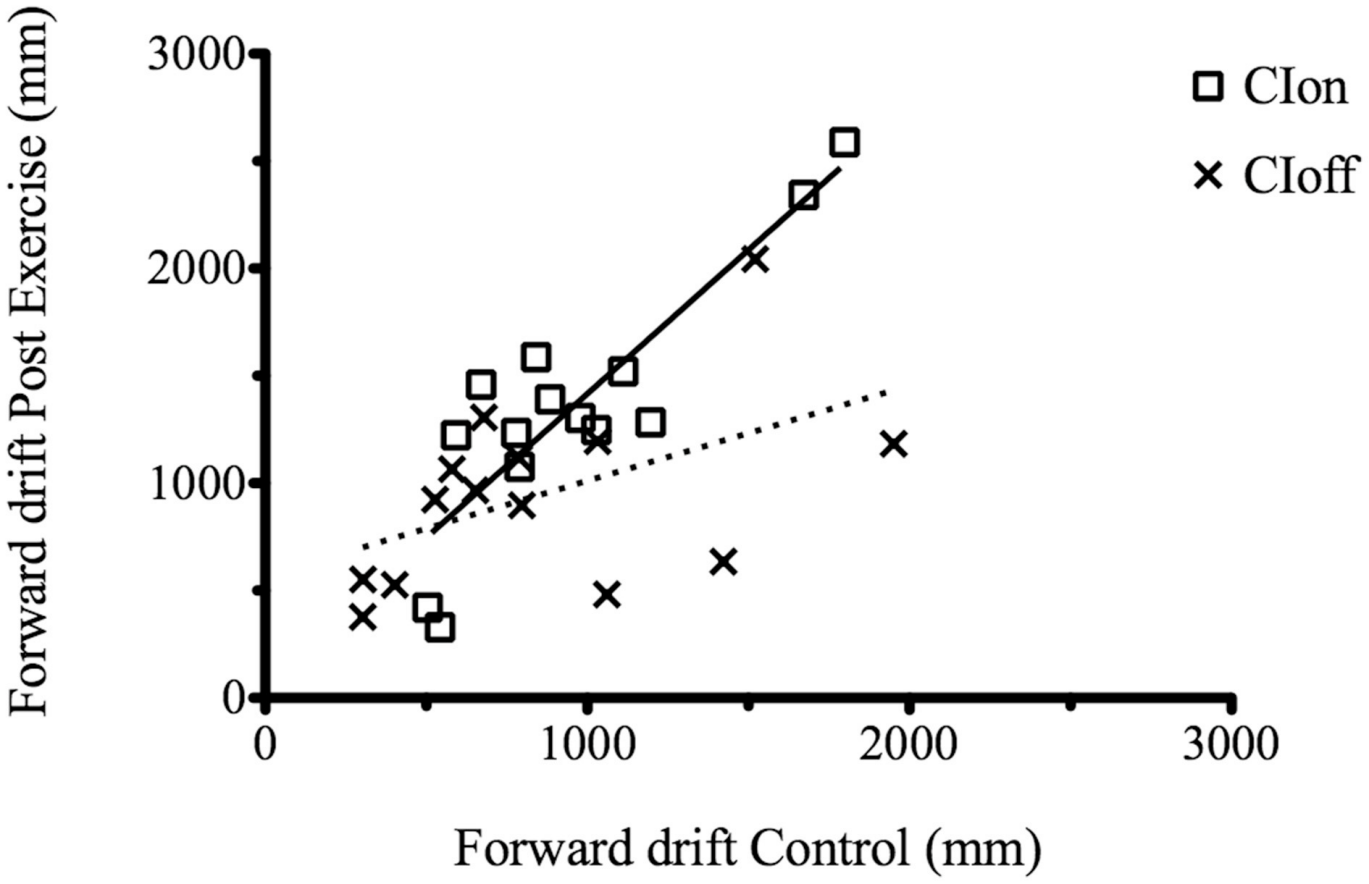
**Correlation between the C and PE forward drift value for the CIon and CIoff conditions**. The dotted line represents the regression for the CIoff and the solid line for the CIon data.

We performed an analysis considering the correlation between the C and PE forward drift value separately for each condition (CIon and CIoff). Results showed a high and significant correlation coefficient for the CIon [*R*^2^ = 0.74, *p* < 0.0001, slope = 1.34] compared to the CIoff (*R*^2^ = 0.2, *p* = 0.06, slope = 0.44) (Figure 2).

This demonstrated that when under the influence of sound, movements are regulated in a consistent manner: the increment in drift after the treadmill experience is consistently proportional across participants but only in the presence of the guidance of sound. All results are summarized in Tables 2, 3.

**Table 2:** **Results in the CIon condition (mean ± standard deviation)**.

	**CIon**			
	**Snow sound**		**Natural footstep sound**	
	**Control**	**Post exercise**	**Control**	**Post exercise**
Forward drift (mm)	965.71 ± 165.38	1448.68 ± 174.75	1091.181 ± 149.62	1553.77 ± 220.33
Step length (mm)	124.59 ± 22.30	153.28 ± 20.78	129.06 ± 22.86	143.29 ± 18.82
Number of steps	20.83 ± 1.35	20.33 ± 1.45	21.16 ± 1.30	21.83 ± 1.70

**Table 3:** **Results in the CIoff condition (mean ± standard deviation)**.

	**CIoff**			
	**Snow sound**		**Natural footstep sound**	
	**Control**	**Post exercise**	**Control**	**Post exercise**
Forward drift (mm)	990.73 ± 251.50	847.16 ± 140.79	895.12 ± 140.21	1214.41 ± 171.44
Step length (mm)	151.61 ± 39.30	154.38 ± 38.55	130.24 ± 19.54	122.30 ± 16.19
Number of steps	18.83 ± 2.85	20.00 ± 1.36	20.33 ± 1.94	21.66 ± 1.40

### Questionnaire results

**Figure 3:**
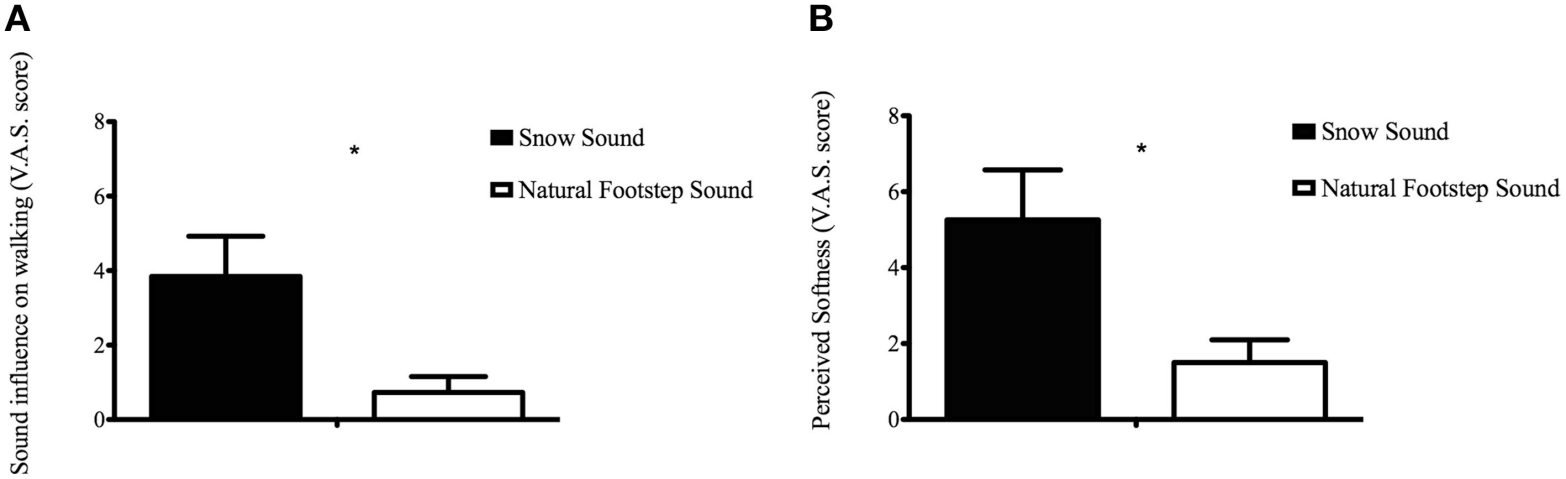
**Visual Analog Scale score of the Sound influence on walking (A) and Perceived softness (B) for each Condition in the CIon Experimental Condition**. ^*^Represents a significance level of *p* < 0.05.

In order to evaluate how participants perceived the surfaces during the CIon Experimental Condition, the VAS results for each questionnaire item were compared between the two surfaces. The analysis conducted with the *t*-test procedure revealed that the SS significantly affected walking when compared to the absence of auditory feedback [*t*_(7.83)_ = 2.681, *p* = 0.028] (see Figure 3A), and significantly affected the perceived softness of the walked-upon surface [*t*_(8.378)_ = 2.599, *p* = 0.03] (Figure 3B). Participants correctly recognized that the typology of the simulated material was aggregate, not solid or liquid. This result is in accordance with the findings reported in our previous study using the same footstep sound engine (Nordahl et al., 2010; Turchet, in press).

## Discussion

In this experiment we investigated whether hearing the sound of footsteps has an impact on the perception-action coupling seen as a typical sensory-motor aftereffect illusion after treadmill walking. With this aim we tested cochlear-implanted patients with their system turned off and on. We also tested whether different sounds, representing different materials, modulated the sensory-motor aftereffect when participants heard their stepping through headphones connected to a pair of sensorized shoes, giving the impression that they were walking on different surfaces. Our results strongly sustain the reliance on auditory feedback: when the cochlear system was turned off, indeed, there was no presence of a sensory-motor aftereffect. In other words, when no sound feedback was available, no increment of drift after experiencing the treadmill walking exercise was present. On the contrary, when the cochlear system was turned on, participants presented an increment in forward drift similar to the one found in normal hearing participants (Turchet et al., 2015). This demonstrates that having a cochlear implant is important not just for language comprehension (Geers et al., 2003) but also for movement planning (Zatorre et al., 2007). Moreover, the results corroborate the fundamental role of sounds in self-movement perception. Studies showed that sounds of action are informative about movement kinematic and dynamic (Young et al., 2013; Cesari et al., 2014; Rodger et al., 2014), and contribute to the internal movement representation (Wolpert et al., 1995). Hence, we found that only combining sound information together with proprioception strengthened the conflict with visual feedbacks (body is moving/body is still), giving rise to the sensory-motor aftereffect. On the other hand, sound feedback removal reduced the amount of available information for the self-movement representation, which in turn lowered the perceptual conflict leading to a lack of sensory-motor aftereffect. In the PE phase of the CIoff condition, indeed, the forward drift was less than the one of the CIon condition, confirming that when sound information is missed, the reduced information conflict experienced during the treadmill walking reduced the aftereffect. Only in the presence of sound did the drift measured before and after the treadmill experience increase consistently and in a stable manner across participants. On the contrary, in the CIoff condition, participants changed their drift after the treadmill walking independently of what they did before. Hence, hearing the step's sound together with proprioceptive information strengthened the conflict with visual feedback: the first two signaling that the body is moving while the latter signaling that the body is still.

Interestingly, contrary to our study, in During and Pelah' s study (Durgin and Pelah, 1999) when the auditory feedback was decreased by means of a pair of earplugs, the sensory-motor aftereffect persisted and even enlarged. It is important to mention though that in During and Pelah's study individuals were asked to run in place while our participants were asked to walk. Even more importantly in During and Pelah's study individuals were able to hear their steps via bone conduction while this was not the case for our participants. These differences, in particular the availability of sound feedback, were able to change the level of perceptual conflict experienced during the treadmill exercise and to show *ad-hoc* motor strategies for maintaining the body balance (Suarez et al., 2007; Cushing et al., 2008; Eustaquio et al., 2011) in particular in the CIoff condition.

Movement regulation by means of sound has been demonstrated by several studies, which showed less sway variability across subjects when sound feedback was delivered during a static balance task (Dozza et al., 2004, 2007) and less movement variability in a dynamic task such as walking (Baram and Lenger, 2012; Rodger et al., 2014). Thus, since in the CIon condition all participants underwent to the same sound feedback (Snow and Natural Footstep Sound), it could be possible that they used it as a reference for movement regulation, which led to a consistent growth of forward drift.

The overall higher forward drift found for NFS compared to SS and the higher number of steps for NFS compared to SS was mainly due to the higher forward drift obtained in the PE phase of the CIon condition. The triple interaction, although marginally significant, revealed, indeed, a lower forward drift only in the CIon condition during the C phase when participants were walking in acoustically simulated deep snow. Those results are also in line with biomechanical studies that showed reduced step cadence and a higher muscle activation when walking on a soft surface compared to a solid one (Pinnington et al., 2005). Moreover, our results sustain that specific movement patterns can be evoked by just listening to the sounds produced (Castiello et al., 2010; Sedda et al., 2011; Turchet and Serafin, 2013; Turchet et al., 2013b, 2015; Rodger et al., 2014), and that the kinematic of a walk can be adjusted according to the heard surface (Turchet and Serafin, 2013; Turchet et al., 2013b, 2015; Rodger et al., 2014), underlining the strong link between sound and action. Sounds are embedded in movement, and every time the sound of an action is heard, brain networks responsible for the execution of that movement are activated (Kohler et al., 2002; Lahav et al., 2007; Aglioti and Pazzaglia, 2010). This mechanism allows the extraction of relevant kinematic action information (Young et al., 2013) and modulates the performed movement accordingly (Young et al., 2013; Cesari et al., 2014).

Kinematic results were supported by the data collected through the questionnaire. Hearing the sound of the snow surface highly influenced the participants' way of walking (Figure 3A) and led them to perceive greater floor softness compared to when no augmented sound was delivered (Figure 3B). This, in line with our previous studies that involved normal hearing individuals (Turchet et al., 2013a,b, 2015), unveils the presence of pseudo-haptic illusions also for the cochlear-implanted population, where the auditory cues successfully created haptic sensations that have no basis in the mechanical signal perceived by the feet. This confirms that even with audio prosthesis that limit the hearable range of frequencies, cochlear implanted individuals were able to recognize the heard surface and use sound as a cue for movement planning.

Taken together, these findings underline that sounds are fundamental for the self-motion perception and prove that auditory feedback is effective in evoking a pseudo-haptic illusion (Turchet et al., 2013b, 2015) that influences the movement kinematic (Castiello et al., 2010; Sedda et al., 2011; Turchet et al., 2013b, 2015; Rodger et al., 2014). In general, we demonstrated that sounds are fundamental not just for language comprehension, but also for movement regulation during a dynamic task, leading to consistent and better movement control when they are available (Dozza et al., 2004, 2007; Baram and Lenger, 2012; Rodger et al., 2014).

This supports the multisensory nature of the sensory-motor aftereffect (Durgin and Pelah, 1999; Turchet et al., 2015), which considers not only proprioception and vision, but also audition; moreover, it breaks new ground on the use of interactive sound feedback in motor learning and opens new questions on how sounds contribute to movement representation in the brain.

## Author contributions

IC Conception, Design of the work, Data acquisition, Data analysis, Data interpretation, Drafting the work, revising the work, Final approval, accountable for all aspects of the work. LT Data acquisition, Data analysis, Revising the work, Final approval, accountable for all aspects of the work. MC Design of the work, Revising the work, Final approval, accountable for all aspects of the work. DM Design of the work, Revising the work, Final approval, accountable for all aspects of the work. PC Conception, Design of the work, Data acquisition, Data analysis, Data interpretation, Drafting the work, revising the work, Final approval, accountable for all aspects of the work.

### Conflict of interest statement

The authors declare that the research was conducted in the absence of any commercial or financial relationships that could be construed as a potential conflict of interest.
